# PB1 mutations as key drivers of influenza A virus evolution

**DOI:** 10.3389/fmicb.2026.1768665

**Published:** 2026-01-29

**Authors:** Canxin Fang, Xin Sun, Yutong Feng, Hong Song, Shengyu Wang

**Affiliations:** 1 Key Laboratory of Infectious Diseases and Biosafety, Education Department of Guizhou Province, Zunyi, China; 2 Department of Microbiology, School of Preclinical Medicine, Zunyi Medical University, Zunyi, China

**Keywords:** evolution, influenza A virus, mutation, polymerase basic protein 1, vaccine

## Abstract

Influenza A virus (IAV) is a zoonotic pathogen with a broad host range, posing an ongoing threat to global public health. As the core subunit of the IAV polymerase, polymerase basic protein 1 (PB1) is essential for viral replication and transcription, and its mutations are key drivers of viral evolution. This review evaluates the impact of PB1 mutations on IAV evolution, with a focus on polymerase activity, host adaptation, transmissibility, and virulence. Additionally, it discusses the implications of these mutations for vaccine development. The review aims to provide insights that can inform influenza surveillance, identify novel antiviral targets, and guide vaccine design.

## 1 Introduction

Influenza A viruses (IAV) are enveloped viruses carrying a single-stranded negative-sense RNA genome. There have been four major influenza pandemics (1918 H1N1, 1957 H2N2, 1968 H3N2, and 2009 H1N1), since the beginning of twentieth century, killing more than 50 million people worldwide, exerting profound impacts on global public health systems, economic stability, and social activities (Ryu and Cowling, 2021; World Health Organization, 2025; Brüssow, 2022). Influenza A virus (IAV) contains a genome with eight single-stranded, negative-sense RNA segments that encode at least 18 proteins (Krammer et al., 2018). Influenza A virus (IAV) RNA-dependent RNA polymerase (RdRp) is a heterotrimer composed of PB2, PB1, and PA, which, together with vRNA and nucleoprotein (NP), forms viral ribonucleoprotein (vRNP) complex to direct the transcription and replication of the viral genome (Wachsmuth-Melm et al., 2025). The PB1 subunit serves as the structural and catalytic core of the RNA polymerase, responsible for replication. Therefore, a mutation at this site can significantly alter viral polymerase activity, host adaptation, and transmissibility (Fodor and Te Velthuis, 2020; Wandzik et al., 2021). Although PB2’s role in cap-snatching and transcription initiation and PA’s role in endonuclease activity are crucial, PB1’s central catalytic role makes it a key target for understanding the mechanistic underpinnings of viral evolution.

PB1 is encoded by viral genomic segment 2 and exhibits a conserved right-handed polymerase fold, primarily comprising three functional subdomains: the Fingers, Palm, and Thumb domains (Peng et al., 2025; Pflug et al., 2017). The Fingers domain binds and positions the viral RNA template, facilitating base pairing between the primer and the template. The Palm domain functions as the catalytic center, where conserved residues within motif A (e.g., D305) chelate magnesium ions to catalyze the formation of phosphodiester bonds. The Thumb domain, in turn, maintains the stability of the RNA polymerase trimer and facilitates the release of RNA products. The template-binding channel (TBC) formed collectively by these three domains features a surface rich in conserved residues, and mutations in this channel can completely abolish enzymatic activity (Fan et al., 2019; Elshina and Te Velthuis, 2021; Te Velthuis et al., 2021; Poon et al., 2012; Te Velthuis et al., 2016; Pflug et al., 2014). Additionally, PB1 contains a priming loop (residues 641–657) that plays a critical role in both the initiation and repositioning stages of viral RNA synthesis (Te Velthuis et al., 2016; Pflug et al., 2014; Samantaray et al., 2023). The N-terminal of the PB1 subunit binds to the C-terminal of the PA subunit, and the C-terminal binds to the PB2 subunit (Stubbs and Te Velthuis, 2014; Zhang et al., 2020; Reuther et al., 2011). These tight interactions ensure the successful assembly and functional integrity of the polymerase complex, which is essential for efficient viral transcription and replication. The collaborative roles of PB1, PB2, and PA within the polymerase complex are fundamental to IAV replication and evolution, and mutations in any of these subunits can have cascading effects on viral fitness.(Wandzik et al., 2020; Keown et al., 2022).

PB1 is the core subunit of the polymerase of influenza A virus. Its main functions in the virus are as follows: promoting the transcription and replication process of the virus: In cap-dependent transcription, PB2 captures the 5′ cap of the host mRNA, which is then cleaved by PA endonuclease to produce primers (Kouba et al., 2019; Noda and Kawaoka, 2010). Subsequently, PB1 catalyzes the extension of the primers to synthesize viral mRNA (Walker and Fodor, 2019; Te Velthuis and Oymans, 2018; Oymans et al., 2018). During the primer-free replication stage, primary replication uses vRNA as a template-dependent initiation loop to *de novo* synthesize cRNA, while secondary replication uses cRNA as a template to internally initiate the synthesis of progeny vRNA (Zhu et al., 2023; Carter and Iqbal, 2024). In this process, the TBC of PB1 ensures the fidelity of synthesis by precisely recognizing the RNA template. PB1 affects the host mechanism through multiple pathways: after forming a heterodimer with PA, it is nuclear import mediated by RanBP5; The CTD domain binding to the host RNA polymerase II enhances the cap capture efficiency (Walker and Fodor, 2019; Krischuns et al., 2021; Li et al., 2023); The interaction with host proteins NUP85 and ArfGAP1 significantly enhances the nuclear entry efficiency and polymerase activity of vRNP (Ling et al., 2022; Peacock et al., 2019). In terms of viral variation and evolution, the high replication error rate of PB1 and the co-evolution of subunits jointly promote antigenic variation and cross-host transmission. Its TBC, initiator loop and interaction interface lay a molecular foundation for the development of novel polymerase inhibitors (Poon et al., 2012). Therefore, PB1 mutations play a key role in influenza virus evolution, and understanding their impact within the context of the entire polymerase complex is crucial for advancing antiviral strategies. This review systematically elaborates on how PB1 mutations influence influenza A virus evolution by regulating polymerase activity, host adaptation, transmissibility, and virulence, and thereby examines the insights and prospects they offer for vaccine development. While PB2 and PA mutations are undeniably significant in IAV evolution, this review prioritizes PB1 due to its core structural and catalytic role within the polymerase complex. Beyond its direct involvement in viral RNA synthesis, PB1 serves as a critical determinant of viral fitness across diverse host environments. This focus is further supported by deep mutational scanning data indicating that PB1 exhibits a relatively lower tolerance for amino acid substitutions compared to PB2 or PA, consistent with its heightened functional constraints within the replication machinery (Li et al., 2023; Günl et al., 2023).

## 2 Influenza A virus PB1 mutations alter the polymerase activity

### 2.1 PB1 mutations that enhance polymerase activity

PB1 mutations can enhance the catalytic efficiency of the polymerase by altering its three-dimensional structure to optimize interactions with substrates or co-factors. For instance, the S524G mutation substitutes serine (S) with glycine (G), reducing side chain volume and polarity. This change likely confers greater conformational flexibility to the PB1 protein, thereby enhancing substrate binding and polymerase activity (Zhang et al., 2021). Similarly, the K577G mutation removes the positively charged lysine, which may alter local electrostatics and potentially enhance viral RNA binding, thereby boosting polymerase activity (Lowen et al., 2019). It is noteworthy that both mutations are situated within the thumb domain, which facilitates the orderly release of the nascent RNA strand from the active site through its interaction with the RNA.

PB1 mutations significantly modulate polymerase activity in a temperature-dependent manner. For instance, G180E/S394P substitutions maintain activity at pyrexic temperatures (40 °C), while the K577E mutation enhances it at 33 °C (Turnbull et al., 2025). Although in-vitro activity is a key fitness determinant, host-level outcomes depend on a multifaceted phenotypic profile. *In vivo*, these temperature-adaptive alterations correlate with high viral titers and severe lung pathology in mice, suggesting that thermal resilience in the polymerase complex contributes to the virus’s ability to overcome host barriers, thereby supporting increased pathogenicity and effective replication (Lowen et al., 2019; Kamiki et al., 2018).

Multisite mutations frequently demonstrate a synergistic impact, notably boosting polymerase activity. The V709I mutation can elevate polymerase activity by 1.3-fold. Introducing multiple mutations (113A/586R/619 N/709I) results in an additional 1.37-fold increase in polymerase activity. Similarly, the A652T mutation alone significantly increased the polymerase activity in A549 and SH-SY5Y cells, while E177G alone did not significantly increase the polymerase activity. However, when the E177G and A652T mutations coexist, the polymerase activity significantly increases in all tested cell lines (Klein et al., 2023). The K198R/D175N double mutation also has a synergistic effect and induces polymerase activity five times higher than that of the wild type in CRNA-directed small genome analysis (Arai et al., 2016). This synergy effect may stem from the interaction between different mutation sites, jointly optimizing the structure and function of the PB1 protein. Multiple mutations may jointly enhance the activity of polymerase by strengthening substrate binding, promoting catalytic processes and increasing interaction with host factors. Table 1 systematically summarizes the PB1 mutation sites associated with enhanced polymerase activity.

**Table 1:** The mutation sites of influenza A virus PB1 and their impact on polymerase activity.

Name	Functional significance
E177G A652T	The polymerase activity was increased, but the transmission ability in ferrets was reduced (Klein et al., 2023)
G180E S394P	The viral polymerase retains its activity, supporting viral replication even at 40 °C (Turnbull et al., 2025).
T182I K198R P627L	A single mutation increases the activity of the polymerase, and when multiple mutations are combined, they produce a synergistic effect to enhance the activity of the polymerase (Arai et al., 2016)
T400S	It enhances the activity of the polymerase, improving the infectivity and replication ability of the virus (Waters et al., 2021)
A469T	Increase the polymerase activity and replication ability of the influenza virus, and promote the spread of the virus in pigs (Wei et al., 2014)
S524G	Enhancing the activity and replication capacity of influenza virus polymerase has improved the aerosol transmission efficiency in ferrets (Zhang et al., 2021)
K577E	Mutation at 33 °C increases the activity and replication ability of polymerase (Kamiki et al., 2018)
K577G	It enhances the activity of the polymerase, improving the infectivity and replication ability of the virus (Lowen et al., 2019)
K578R	It increases polymerase activity (Li et al., 2023)
P596S L598P	It increases polymerase activity (Welkers et al., 2019)
V709I	It enhances the activity of the polymerase, improving the infectivity and replication ability of the virus (Guo et al., 2023)
R239	Reduce the activity of viral polymerase (Xu et al., 2021)
S261N	Reduce the activity of viral polymerase (Chin et al., 2016)
L319Q	Reduce the activity of viral polymerase, detoxify, and be sensitive to temperature (Nogales et al., 2022)
A648 H649 G650 P651	Reduce the activity of viral polymerase (Te Velthuis et al., 2016)
K612R	The replication ability of the virus has significantly declined (Li et al., 2021)
K229R	Reduce the activity of viral polymerase (Goldhill et al., 2018; Goldhill et al., 2021)

### 2.2 PB1 mutations that inhibit polymerase activity

Mutations at the PB1 site substantially diminish polymerase activity by disrupting a critical domain of the PB1 protein. For example, the activity of the polymerase may be related to the size of the hydrophobic side chain at position 362. When M362 mutates to other hydrophobic residues, the activity of the polymerase decreases, while that of the charged residues becomes inactive. The polymerase activity of mutants such as 362I and 362V is reduced (Chan et al., 2023). The L319Q mutation replaces leucine (L) with glutamine (Q), introducing a polar side chain that may disrupt the hydrophobic core of the PB1 protein, leading to a decrease in polymerase activity (Cox et al., 2020; Cox et al., 2016; Nogales et al., 2022). This mutation is located in the finger domain and may have affected the structure and function of the finger domain.

Partial site mutations weaken the activity of the polymerase by reducing the binding ability of the PB1 protein to substrates or cofactors. For instance, R239 is a key amino acid residue located in the conserved sequence F, which is crucial for maintaining the transcriptional activity of the RdRp of influenza A virus. The mutation replaces arginine (R) with uncharged amino acids such as serine, glutamine, glycine, alanine and leucine, completely losing the polymerase activity. This might be because the positive charge at the R239 site is crucial for maintaining the structure and function of the PB1 protein (Xu et al., 2021).

The initiator loop on PB1 is situated at amino acid positions 641–657, forming a β hairpin structure that extends from the thumb subdomain to the polymerase active site. A mutant lacking four conserved tip residues (A648, H649, G650, and P651) of the PB1β hairpin, termed *Δ*648-651, was created. Experimental findings revealed a notable decrease in RNA synthesis efficiency for this mutant in both cell culture and *in vitro* settings, particularly during the terminal initiation process, resulting in a tenfold activity reduction. This underscores the critical role of these four residues in PB1β hairpin function. Additional mutants were generated by altering a single amino acid at the tip of PB1β hairpins, including H649A, G650A, P651A, and P647A. These mutants exhibited impaired terminal *de novo* activation activity, with the P651A mutant showing the most significant impact, suggesting the crucial involvement of the P651 residue in facilitating glycosyl-base support for initiating NTP. Moreover, a mutant [Δ (648–651)] lacking the four conserved residues (A648, H649, G650, and P651) at the β hairpin’s end was developed. RNP reassortment experiments demonstrated a substantial reduction in viral RNA synthesis efficiency (vRNA, cRNA, and mRNA) in cell culture for this mutant, highlighting the essential role of the PB1 β hairpin structure in the efficient RNA synthesis of influenza A virus (Te Velthuis et al., 2016).

Site mutations can reduce the activity of polymerase by affecting the SUMO modification of PB1 protein. Although the SUMOylation modification at the K612 site of PB1 does not change the protein half-life or subcellular localization, it is crucial for maintaining its viral RNA binding ability. After the K612R site mutation eliminated the Sumoylation modification, the virulence of the virus in the mouse model decreased by more than 90%, and it completely lost its airborne transmission ability in the ferret model, fully demonstrating the irreplaceable role of this post-translational modification in the spread of the virus (Li et al., 2021).

### 2.3 PB1 mutations in influenza antiviral development

The polymerase is highly conserved across influenza A subtypes, suggesting that these sites could be effective targets for potential influenza antiviral development. RdRp inhibitors are a key focus of anti-influenza drug development, with clinical or approved agents targeting all three subunits (Li et al., 2025). The PB1 inhibitor favipiravir, licensed in Japan in 2014 for refractory or emerging influenza, blocks RNA elongation by chain insertion and demonstrates broad activity with minimal resistance. However, its prolonged high-dose regimen and teratogenic liability highlight the need for next-generation alternatives (Joshi et al., 2021; Baranovich et al., 2013).

The emergence of resistance to favipiravir in the influenza virus is constrained by a high genetic barrier. Resistance requires a specific mutation at residue K229 in the PB1 subunit (K229R), located within the highly conserved polymerase motif F and critical for stabilizing nucleoside triphosphate (NTP) binding. This mutation severely impairs polymerase activity, leading to a dramatic reduction in viral replication fitness. To overcome this, the virus must also acquire a compensatory mutation, P653L, in the PA subunit, which restores polymerase function. The stringent requirement for these two concurrent mutations establishes a formidable genetic obstacle, making the development of resistance to PB1-targeting inhibitors clinically challenging. This also underscores the functional integration of the RdRp complex, where cooperative interactions among its subunits are essential for maintaining viral replication and overall viral adaptability (Goldhill et al., 2018; Goldhill et al., 2021). Representative mutation sites known to impair polymerase activity are listed in Table 1.

## 3 PB1 mutations affect the host adaptability and transmission ability of influenza A virus

### 3.1 PB1 mutations that facilitate the host adaptability

The natural reservoir of the influenza A virus is wild birds, although it also infects mammals. The primary subtypes associated with human infections include H1, H2, H3, H5, H7, and H9; however, only H1, H2, and H3 are consistently found within the human population. Cross-species transmission of the virus encounters multiple barriers but occasionally overcomes these obstacles to generate new lineages. Avian influenza viruses, such as the highly pathogenic avian influenza H5N1, may sporadically enter human populations but typically do not spread easily among individuals. Nevertheless, the potential for the establishment of new lineages remains. Poor polymerase activity is one of the main obstacles it faces in mammalian cells. There is evidence suggesting that the virus overcomes this obstacle by acquiring PB1 host-adaptive mutations. A wide range of polymerase-adaptive mutations can work together to overcome this defect. Identifying and monitoring such emerging adaptive mutations is of great significance for assessing the pandemic potential of avian influenza viruses (Williams et al., 2024; Wang et al., 2020). Avian influenza virus polymerase has low activity in mammalian cells, which limits viral replication. However, various adaptive mutations can enhance its activity, such as PB1 mutations, etc. (Wang et al., 2020; Li et al., 2004). Polymerase subunit mutations that increase transcriptional activity are the basis for avian influenza virus adaptation to human hosts (Taubenberger et al., 2005; Xu et al., 2012).

While entire avian-origin polymerase complexes typically exhibit restricted activity in mammalian cells due to host-species barriers, the strategic incorporation of avian PB1 fragments contributed significantly to the 1918, 1957, and 1968 pandemics (Wendel et al., 2015; Worobey et al., 2014). Mutation N375S was present among the amino acid PB1 sequences in 1918, 1957 and 1968 (Williams et al., 2024). Studies have shown that in luciferase reporter gene assays, replacing human-derived PB1 with avian PB1 significantly increased polymerase activity. This enhancement may have improved the transcription and replication efficiency of the viral genome in human cells. Compared with avian precursors, the avian PB1 of the 1968 pandemic virus contains three amino acid substitutions (K121R, L4V, R327K) (Williams et al., 2024). These mutations further enhanced the activity of the polymerase and the adaptability of the virus in the host. Studies have also shown that the PB1 protein of the influenza virus plays a critical role in the replication of the viral genome within infected cells, and is a key factor in determining the virus’s temperature sensitivity (Turnbull et al., 2025; Li et al., 2009). Viruses with avian-like PB1 are capable of tolerating high temperatures associated with fever, whereas human-derived influenza viruses exhibit significant temperature sensitivity at febrile temperatures. Avian-derived PB1, or even a few critical mutations, can confer high-temperature tolerance to the virus. This not only explains why certain avian-origin or pandemic viruses cause more severe disease in humans but also highlights the central role of PB1 in determining host adaptability (Turnbull et al., 2025).

When the 2009 pandemic broke out, the serine residue related to birds at position 216 of PB1 was replaced with glycine residue related to humans, which was a powerful evolutionary adaptation that affected the currently globally prevalent seasonal human H1N1 virus (Lin et al., 2019). Mutations at the PB1 site may have enhanced host adaptability, a situation that could explain why the two recent pandemics in humans had avian-like sources of PB1 protein (Taubenberger et al., 2005). Adaptive mutations in the PB1 gene are critical for influenza viruses to overcome host barriers. The K586R and D619N substitutions in PB1, which emerged in Asia around 2002 before spreading globally, are hypothesized to have enhanced viral fitness. The temporal alignment of these mutations with the 2002 large-scale H3N2 epidemic suggests they may have provided a selective advantage. Rather than acting as sole drivers, such site-specific mutations likely optimized polymerase activity or stability, thereby contributing to the broader ecological success and prevalence of seasonal influenza strains during that period (Klein et al., 2023; Sun et al., 2021; Gíria and Rebelo de Andrade, 2014).

The PB1-K577E mutation serves as a mammalian-adaptation hallmark: it maximizes polymerase activity at 33 °C for upper-airway expansion while retaining high function at 37 °C, enabling onward replication in the lower respiratory tract and lethal murine disease (Kamiki et al., 2018). When there is a lack of avian influenza viruses with effective transmissibility, the PB1-Q621R and NP-R351K mutations increase viral replication and transmission in piglets (Su et al., 2021). The T296R mutation expands the tissue tropism of the virus, enabling it to replicate in the brain of mice and reflecting its deep adaptation to mammalian hosts (Yu et al., 2015). It is worth noting that host adaptive mutations often exhibit the characteristic of “functional synergy.” The mutations L298I, R386K, and I517V act synergistically to improve the adaptability of the A(H1N1)pdm09 virus during the initial infection phase by enhancing the stability of the PB1 protein or interacting with other polymerase subunits (PB2, PA) (Santos et al., 2023). Additionally, I368V may synergistically interact with established mammalian adaptive mutations like A588V and K702R in the PB2 gene, thereby augmenting the virus’s adaptability in human hosts (Chen et al., 2025; Xue et al., 2025). The PB1 mutations associated with host adaptation are compiled in Table 2.

**Table 2:** The mutation of influenza A virus PB1 and their influence on host adaptability and viral transmission ability.

Name	Functional significance
L13P	The enhancement of the influenza virus polymerase activity and replication capacity increases the pathogenicity and transmissibility of the virus (Dreier et al., 2019)
T123A	Enhanced replicative fitness (Pauly et al., 2017).
T296R	Enhanced polymerase activity and replication capacity of the influenza virus contribute to improved fitness in mammalian hosts (Yu et al., 2015)
S524G	Enhanced activity and replication capacity of influenza virus polymerase increases aerosol transmission efficiency in ferrets (Zhang et al., 2021)
K577E	Mutation at 33 °C increases the activity and replication ability of polymerase (Kamiki et al., 2018)
K586R D619N	Increase the activity and replication capacity of influenza virus polymerase (Sun et al., 2021)
Q621R	The combined mutation with NP-R351K increases the replication and transmission ability of avian influenza virus in pigs (Su et al., 2021)
H99Y	The mutation enabled the virus to acquire the ability to spread through the air among ferrets (Linster et al., 2014)
L675A N676Q	Reduce the transcriptional activity of influenza virus polymerase and damage the spread of influenza virus (Li et al., 2025)
D445G S444P	Reduce the activity of viral polymerase (Poon et al., 2012)
N306T D439E	Reduce the activity of viral polymerase (Poon et al., 2012)
T156A F740L	The individual T156A and F740L mutations had no effect on the activity of viral RNA polymerase. When co-mutated with PA E349G, the replication ability in mouse cells was significantly enhanced, and the release of infectious viral particles increased tenfold (Slaine et al., 2018)

### 3.2 The impact of PB1 mutations on viral transmission

Influenza viruses can propagate through the air, primarily via respiratory droplet transmission or aerosol transmission, which serves as a crucial mechanism for the transfer of these viruses from animals to humans. Mutations at specific sites within the PB1 gene can markedly alter the efficiency of aerosol transmission in influenza viruses. For example, the S524G and L13P mutations illustrate distinct effects on disease transmission. In the ferret model, the S524G mutation enhances aerosol transmission efficiency (Zhang et al., 2021), thereby facilitating the spread of the virus. Conversely, the L13P mutation not only increases pathogenicity but also improves contact transmission capacity among guinea pigs (Dreier et al., 2019). The H99Y mutation: The H99Y mutation on PB1 is one of the key mutations for the AH5N1 virus to achieve airborne transmission among ferrets. In ferret passage experiments, the H99Y mutation emerged as a secondary variant in the early passage and then became the dominant strain, indicating that it gained an evolutionary advantage by enhancing its airborne transmission ability. When the H99Y mutation coexists with other key mutations, such as certain mutations on the PB2-E627K and HA genes, the virus can be transmitted through the air among ferrets. However, if the H99Y mutation is absent, the virus cannot be effectively transmitted among ferrets even if other mutations are present (Linster et al., 2014).

However, not all mutations in the PB1 gene are beneficial for virus transmission. Some mutations at the PB1 L675/N676 locus can seriously impair the transmission of the influenza virus. Researchers have found that replacing L675 and N676 with other amino acids (such as L675A/N676A) significantly affects the transcriptional activity of influenza virus polymerase, especially the synthesis of the poly(A) tail. These mutations lead to A reduction in the synthesis of poly(A) tails of viral mRNA, thereby affecting the replication and spread of the virus. After infecting cells, the mRNA expression levels, protein levels and plaque area of viruses carrying these mutations are significantly lower than those of wild-type viruses, resulting in a decrease in the transcriptional and transmission efficiency of the viral genome (Li et al., 2025).

The above-mentioned mutations are mostly concentrated in the functional domains of the PB1 protein (such as the polymerase active center or the PB2 binding domain), and their amino acid substitutions may affect the viral transmission phenotype by altering the protein conformation or interaction network. An in-depth study of the impact of PB1 gene mutations on the transmission capacity of influenza viruses helps us better understand the transmission mechanism of viruses and provides a theoretical basis for the prevention and control of influenza viruses. By monitoring the changes in these key mutation sites, potential changes in the virus’s transmission capacity can be warned in advance, and corresponding prevention and control measures can be taken. The effects of PB1 mutations on transmission are compiled in Table 2.

## 4 The role of PB1 mutations in regulating influenza A virus virulence

### 4.1 PB1 mutations enhancing viral virulence

Combined mutations such as K198R/D175N: In mouse models, viruses carrying mutations such as K198R and K198R/D175N lead to more severe weight loss and reduced survival rates. Compared with the wild-type virus, the viral titers of these mutant viruses in the lungs of mice increased by 130 times (on the 3rd day after infection) and 550 times (on the 6th day after infection), respectively, and the pathological damage was exacerbated, suggesting that these mutations significantly increased virulence by enhancing the viral replication ability. These mutations located in the β-ribbon domain of the vRNA interaction region may alter the spatial conformation and binding ability of PB1 to the vRNA promoter, thereby facilitating the formation of more stable complexes and affecting viral replication and virulence (Arai et al., 2016).

In chickens, M317V mutation significantly enhances the activity of viral polymerase at 37 °C, promoting the replication efficiency of the virus within host cells, leading to accelerated viral replication, increased mortality, and shortened time to death (Youk et al., 2021). V113A/K586R/D619N/V709I (Background: HK/68) This mutation combination induced immune infiltration and bronchial thickening in the lungs of mice, resulting in a weight loss of up to 17% in mice, accompanied by elevated levels of inflammatory factors such as IL-6, ISG-15 and IFN-β, indicating that it enhances pathogenicity by strengthening viral replication and inflammatory responses (Sun et al., 2021). The PB1-V719M substitution, in combination with PB2-E627K, markedly enhances the pathogenicity of H7N9 avian influenza virus in mice (Wang et al., 2024).

In the mouse model, the PB1 mutant Q687R virus of the A/South Africa/3626/2013 strain exhibited high pathogenicity, with an LD₅₀ value of 5.0 log₁₀ EID₅₀/mL, which falls within the high-pathogenicity threshold defined in the study. Moreover, A high viral RNA copy number could be detected in the lung tissue of mice on the third day after infection, indicating a strong replication ability of the virus in mice. The Q687R mutation in the PB1 gene of the virus may enhance the stability of the polymerase complex, thereby increasing its activity. The Q687R mutation is located at the C-terminal of the PB1 protein. The C-terminal of the PB1 protein (residues 678–757) forms a binding site that tightly binds to the N-terminal of the PB2 protein, which is crucial for maintaining the stability and activity of the polymerase complex. The Q687R mutation may enhance the stability of the polymerase complex by altering the interaction between PB1 and PB2, thereby increasing its activity, promoting the replication and transcriptional efficiency of the virus within the host cell, enabling the virus to proliferate more rapidly and thus enhancing the pathogenicity of the virus (Al Farroukh et al., 2022). Virulence-enhancing PB1 mutations are cataloged in Table 3.

**Table 3:** The mutation sites of influenza A virus PB1 and their influence on viral virulence.

Name	Functional significance
V113A	Increase the activity and replication capacity of influenza virus polymerase (Sun et al., 2021)
T182I K198R P627L	A single mutation increases the activity of the polymerase, and when multiple mutations are combined, they produce a synergistic effect to enhance the activity of the polymerase (Arai et al., 2016)
M317V	The polymerase activity of the virus in chickens increases at 37 °C, leading to accelerated virus replication and enhanced pathogenicity (Youk et al., 2021)
K586R D619N	Increase the activity and replication capacity of influenza virus polymerase (Sun et al., 2021)
Q687R	It enhanced the stability and activity of the polymerase, thereby explaining the high pathogenicity of the virus (Al Farroukh et al., 2022)
Q694H I695K	The replication ability of the virus in avian cells has been enhanced, enabling the virus to better adapt to the avian environment (Ran et al., 2022)
L319Q	Reduce the activity of viral polymerase, detoxify, and be sensitive to temperature (Nogales et al., 2022)
G622D	The activity of polymerase is reduced by 40 times, lowering the virulence of the virus (Feng et al., 2016)
V43I	Increase replication fidelity and reduce viral pathogenicity (Naito et al., 2017; Cheung et al., 2014)
D193G K197E	The virulence of the virus has been reduced (Du et al., 2016)
V709L	Increase the activity and replication capacity of influenza virus polymerase (Sun et al., 2021)
K198A M199A I200A	Reduce the activity of viral polymerase (Uchikubo-Kamo et al., 2024)

### 4.2 PB1 mutations attenuating viral pathogenicity

Compared with the wild-type virus, the single V43I (H5N1) mutation reduced the polymerase activity by approximately 40%. The wild-type virus has a greater replication advantage than the V43I mutant, whose pathogenicity to mice is significantly reduced. The V43I mutation is located within the presumed viral RNA binding domain at the N-terminal of PB1 and is usually quite conserved (Naito et al., 2017). The PB1-L319Q/PB2-265S combination: A single mutation can reduce the virus’s virulence by 10 times, while a combined mutation enhances the virulence by 20,000 times and completely block transmission in ferrets and guinea pig models, suggesting the key role of synergistic effects in virulence reduction (Cox et al., 2020). Triple mutation I298L/K386R/V517I: The viral infectious titer is significantly lower than that of the wild type, and the inhibitory effect of the triple mutant is the strongest (Santos et al., 2023).

Q694H and I695K: They enhanced the replication ability of the virus in avian cells, but the viral titers of the viruses carrying these mutations in the lungs of mice were significantly reduced, indicating that these mutations decreased the virulence of the virus in mammalian cells and mice. Significantly reducing the titer of the virus in the lungs of mice indicates its ability to inhibit replication within mammalian cells. As the mutation is located in the C-terminal part of PB1 and affects the growth of the virus in mammalian cells, it is speculated that it may be related to the function of the polymerase (Ran et al., 2022).

Site mutations impact nuclear import and consequently affect the virulence of the virus. The D193G and K197E mutations reside within the β-folding region of the PB1 protein, significantly impairing the viral replication capacity. Research indicates that these mutations can result in a substantial reduction in virus production. The underlying mechanism involves the disruption of the interaction between PB1 and RanBP5, which adversely affects the nuclear import function of the PB1 protein. Experiments have shown that mutating these key residues (such as D193G and K197E) can lead to a significant reduction in viral yield and affect nuclear import by interfering with the PB1-RanBP5 interaction (Du et al., 2016). The G622D mutation replaces glycine (G) with aspartic acid (D), hindering the binding of PB1 protein to viral RNA and reducing the activity of polymerase, thereby weakening the virulence of the virus in mice (Feng et al., 2016).

The virulence of the influenza A virus, a critical factor in its pathogenicity, is intricately regulated by the virus’s genomic variation and evolutionary processes. Site-specific mutations in the PB1 protein have been shown to significantly influence the virus’s virulence. Recent research indicates that these mutations affect virulence through various mechanisms, including the regulation of viral polymerase function, the modulation of nuclear import of viral proteins, and the alteration of host adaptability. Specifically, the PB1 protein can exert bidirectional regulation of viral virulence through mutations at distinct sites. Mechanisms that enhance virulence primarily involve mutations in the β-ribbon domain (e.g., K198R/D175N), which improve replication efficiency by optimizing the vRNA promoter binding conformation. Additionally, mutations at the C-terminal of PB1 (e.g., Q687R) enhance polymerase activity by stabilizing the interaction interface between PB1 and PB2. Conversely, attenuated mutations occur through different pathways: V43I diminishes activity by disrupting the conformation of the RNA binding domain, while D193G/K197E impedes vRNP nuclear import by interfering with its interaction with RanBP5. These studies systematically elucidate the diverse molecular mechanisms by which PB1 site mutations regulate viral virulence, offering new strategic targets and a theoretical foundation for vaccine optimization and antiviral drug development. Attenuating PB1 mutations are also cataloged in Table 3.

## 5 The application of influenza A virus PB1 mutations in vaccine development

Influenza vaccination remains one of the most effective strategies for preventing and controlling viral transmission, significantly reducing infection rates and associated complications (Bosaeed and Kumar, 2018; Cowling and Okoli, 2024). Commonly used influenza vaccines include inactivated influenza vaccines (IIV), live attenuated influenza vaccines (LAIV), and recombinant influenza vaccines (RIV) (Kim et al., 2022; Grohskopf et al., 2025). Due to the lack of proofreading activity in their RNA polymerase, influenza viruses exhibit genetic instability, leading to frequent antigenic mutations that drive antigenic drift (Boivin et al., 2010; Houser and Subbarao, 2015). This viral characteristic, combined with production challenges (e.g., mutations arising from egg-based cultivation and prolonged manufacturing cycles), contributes to the suboptimal effectiveness of current vaccines (Hannoun, 2013; Raymond et al., 2016; Scott et al., 2025). Accumulating studies suggest that mutations in the PB1 gene may contribute to enhancing the protective efficacy and safety of LAIVs by optimizing viral attenuation and regulating replication fidelity.

### 5.1 Regulation of viral polymerase activity and fidelity by PB1 mutations: implications for LAIV development

Multiple PB1 mutations modulate influenza virus replication by altering the activity and fidelity of the viral RdRp, thereby influencing mutation frequency, genetic stability, and vaccine safety. Such effects are particularly relevant to the development of LAIV, where attenuation relies on controlled viral replication and constrained viral evolution rather than complete inhibition of viral replication. In contrast, fidelity-based strategies are not applicable to non-replicating vaccine platforms (e.g., inactivated, subunit, or mRNA vaccines) (Zhou et al., 2016). The PB1-V43I mutation enhances nucleotide selectivity of the RdRp complex and increases polymerase fidelity without substantially impairing elongation activity or overall viral replication in certain IAV backgrounds. Compared with wild-type PB1, the V43I mutant exhibits an approximately 1.9-fold reduction in mutation frequency and improved nucleotide incorporation accuracy *in vitro*, contributing to enhanced genetic stability during vaccine production and serial passage. Preservation of replication competence permits sufficient *in vivo* replication to elicit robust immune responses, while reduced mutation rates limit within-host diversification and adaptive potential under vaccine-related replication conditions, supporting its relevance for LAIV design (Naito et al., 2017; Cheung et al., 2014). Notably, polymerase fidelity is a property of the entire RdRp complex, rather than PB1 alone.

K235R serves as a crucial regulatory site in the RNA polymerase of the influenza virus, with its mutation significantly enhancing the polymerase’s replication fidelity. The K235R mutation increases the incorporation rate of correctly paired nucleotide triphosphate (NTP) by RNA polymerase by a factor of 1.9, while simultaneously decreasing the incorporation rate of incorrectly paired NTPs. This alteration results in an enhancement of transcriptional fidelity by approximately 4.6 times. Critically, this mutation represents a breakthrough as it enhances both fidelity and activity, overcoming the traditional “fidelity-activity trade-off” often seen in high-fidelity variants. This mechanism elucidates the unique role of K235R in optimizing RNA polymerase function and positions it as a potential target for the design of structure-based influenza virus vaccines and the development of antiviral drugs. Thus, it provides a theoretical foundation for the creation of high-fidelity vaccines and innovative therapeutic strategies (Xu et al., 2021).

The PB1-L66V mutation further underscores the importance of polymerase fidelity. It prevents the reversion of the temperature-sensitive (TS) phenotype during serial passage, a critical factor for LAIV safety. By limiting mutation accumulation during replication, L66V reduces the risk of virulence restoration or antigenic drift, thereby enhancing the long-term genetic stability of attenuated vaccine strains (Mori et al., 2021). The PB1-Lys471 mutation in the influenza A virus RNA polymerase has been engineered to confer a TS phenotype, a critical feature for the development of genetically stable LAIVs. Among various substitutions, the PB1-K471P mutant maintained the TS phenotype across serial passages without reverting to wild-type. This variant replicated efficiently at lower temperatures (31 °C–34 °C) but was severely restricted at 37 °C, mimicking the temperature gradient of the human upper respiratory tract. These characteristics enhance the genetic stability and safety of LAIVs by minimizing the risk of reversion to virulence. Importantly, PB1-K471P elicited robust immune responses, including significant IFN-γ production and high hemagglutination inhibition antibody titers, offering broad protection against lethal influenza challenges in animal models. These findings underscore the potential of PB1-Lys471-based mutations, particularly K471P, as a stable and immunogenic backbone for next-generation LAIVs effective against both seasonal and emergent influenza strains (Naito et al., 2025).

### 5.2 PB1 mutations associated with viral adaptability and their platform-specific application

Adaptive mutations in PB1 play essential roles in host adaptation, cross-species transmission, and viral fitness, which can be strategically employed to optimize vaccine seed strains in a platform-specific manner. Mutations that enhance viral fitness are beneficial for IIV production, where the focus is on maximizing antigen yield (Santos et al., 2023). However, such mutations are generally contraindicated for LAIV, where controlled replication and constrained viral evolution are essential (Cobbin et al., 2013).

For example, the PB1 mutations L298I, R386K, and I517V enhance viral replication and hemagglutination titers in mammalian cells, reflecting the virus’s adaptation to human hosts. These mutations are useful in the context of IIV production to increase antigen yield but are unsuitable for LAIV, as they may promote uncontrolled viral replication and heighten the risk of viral evolution during vaccine propagation (Santos et al., 2023; Bai et al., 2024). This principle extends to vaccine seed engineering using reverse genetics. Incorporation of a homologous PB1 gene from the target strain into a 5:3 reassortant constellation (donor HA, NA, and PB1 in a high-growth backbone such as A/Puerto Rico/8/34) consistently improves viral growth and antigen yield compared with conventional 6:2 reassortants, underscoring PB1’s role in optimizing genomic compatibility during vaccine production (Almeida et al., 2022; Gíria et al., 2016).

Conversely, adaptive mutations like PB1-Q694H and I695K, identified in bat-derived influenza viruses, enhance replication in avian cells but significantly restrict growth in mammalian cells and mice (Ran et al., 2022). These mutations may offer a strategy for optimizing LAIV safety while preserving immunogenicity by enhancing replication in specific hosts. These examples highlight the importance of selecting platform-specific mutations. The mutations suitable for IIV production differ fundamentally from those required for LAIV attenuation, underscoring the necessity of precise mutation-platform matching in vaccine design.

### 5.3 Regulation of viral virulence by PB1 mutations and their value as vaccine targets

PB1 mutations significantly influence influenza virus virulence through several mechanisms, including temperature sensitivity, modulation of polymerase fidelity, and disruption of viral RNA interactions. These properties offer distinct mechanisms that can be exploited in the development of LAIV.

For example, the PB1-L319Q mutation induces a temperature-sensitive phenotype, restricting viral replication at elevated temperatures (37 °C–39 °C) while allowing replication at lower temperatures typical of the upper respiratory tract. This temperature sensitivity enables sufficient local replication to induce protective immunity while limiting infection in the lower respiratory tract, which is crucial for LAIV safety. Notably, the L319Q mutation works synergistically with the PB2-N265S mutation to further reduce viral replication, pathogenicity, and transmissibility, emphasizing the importance of combinatorial attenuation strategies for LAIV development (Cox et al., 2020; Nogales et al., 2022).

Other PB1 mutations, such as Y82C and G622D, reduce polymerase fidelity or impair vRNA binding, respectively, leading to attenuation through distinct mechanisms. The PB1-Y82C mutation reduces polymerase fidelity to induce a mutator phenotype, characterized by a 2.3-fold higher nucleotide misincorporation rate and 1.9–3.5-fold elevated mutation frequency *in vitro*, which ultimately confers significant *in vivo* attenuation (Naito et al., 2019). The G622D mutation impairs PB1-vRNA binding, causing a substantial reduction in polymerase activity and virulence, making it a potential target for attenuated vaccine design, particularly for zoonotic influenza strains like H5N1 (Feng et al., 2016).

These findings demonstrate the complexity of PB1 mutations in regulating viral virulence and highlight the necessity for rational design strategies that leverage these mutations to develop safe and effective LAIVs. It is important to note that all of these effects occur within the context of the RdRp complex, where PB1 role is modulated by interactions with PB2, PA, and vRNA.

Utilizing PB1 mutations to attenuate influenza viruses for LAIV development raises several evolutionary risks that must be carefully managed. These risks include the potential for reversion to a more virulent phenotype, the accumulation of compensatory mutations, and the possibility of reassortment with wild-type viruses. Reversion to virulence is particularly concerning for mutations like L319Q, which, although effective in reducing virulence, may be subject to selective pressure during vaccine propagation, leading to the recovery of virulence. Similarly, compensatory mutations, such as those observed with PB2-N265S in combination with L319Q, may restore replication and virulence, emphasizing the need for monitoring such mutations during vaccine production and passage. Reassortment, especially in adaptive mutations such as L298I, could lead to the introduction of high-fitness genotypes into the wild-type virus, potentially enhancing its cross-species transmission. To mitigate these risks, employing multiple-site mutations (e.g., L319Q combined with other mutations) provides a more robust attenuation strategy, minimizing reliance on any single mutation and reducing the likelihood of reversion or compensatory mutations. Table 4 provides an overview of vaccine design strategies that exploit PB1 mutations, particularly those regulating polymerase fidelity or enabling attenuation.

**Table 4:** Summary of key PB1 mutations for influenza A virus vaccine development.

Name	Vaccine platform	Experimental evidence (*in vitro* / *in vivo*)	Genetic stability across passages	Key findings & potential application
V43I	LAIV	In vitro: Reduces mutation frequency by 1.9-fold and increases nucleotide incorporation accuracy.	Enhances genetic stability during production and serial passage.	Relevant for LAIV design to improve genetic stability (Naito et al., 2017; Cheung et al., 2014).
K235R	LAIV	*In vitro*: Increases correct NTP incorporation rate by 1.9 - fold and transcriptional fidelity by ~4.6 – fold.	Not explicitly reported.	Potential target for structure-based vaccine development (Xu et al., 2021).
L66V	LAIV	Phenotypic study: Prevents reversion of the TS phenotype during serial passage.	High; maintains the TS phenotype and prevents reversion to wild-type.	Increases polymerase fidelity, thereby improving the genetic stability of LAIV seed strains and reducing reversion risk (Mori et al., 2021).
K471P	LAIV	*In vitro*: TS phenotype >100-fold reduction in polymerase activity at 37 °C. *In vivo*: Protects mice from lethal PR8.	High; maintained TS phenotype across ≥5 passages; no reversion.	Stable TS backbone for high-yield LAIVs; applicable to seasonal and pre-pandemic vaccines (Naito et al., 2025).
L298I, R386K, I517V	IIV	*In vitro* / *in vivo*: Enhances viral replication and hemagglutination titers in mammalian cells. Adaptive mutations likely improve production fitness.	Not explicitly reported.	Useful for optimizing IIV seed strains to increase antigen yield. Not suitable for LAIV (Santos et al., 2023; Bai et al., 2024).
Q694H, I695K	LAIV	*In vivo* / *in vitro*: Enhances replication in avian cells but severely restricts growth in mammalian cells and mice	Stable in avian cells; no reversion reported.	Provides a host - range restriction strategy for designing safer LAIVs that maintain immunogenicity while enhancing safety (Ran et al., 2022).
L319Q	LAIV	*In vivo*: Reduces viral replication and pathology in ferrets; decreases transmissibility in guinea pigs.	Single mutation carries reversion risk; stability improved in combination with other mutations.	Confers a temperature - sensitive phenotype. A key attenuation target for LAIV. Recommended for use in combination for robust attenuation (Cox et al., 2020; Nogales et al., 2022).
Y82C	LAIV	*In vitro*: 2.3-fold higher nucleotide misincorporation rate. *In vivo*: 10-fold lower EID₅₀ in embryonated eggs.	Moderate; prone to excessive mutation accumulation; requires passage monitoring.	Novel LAIV attenuation via mutator phenotype; suitable for zoonotic/pandemic strains (Naito et al., 2019).
G622D	LAIV	*In vivo*: Attenuates mouse pathogenicity by 500-fold. *In vitro*: Reduces polymerase activity by 40-fold.	Not explicitly reported.	A potential target for LAIV development against highly pathogenic strains (e.g., H5N1) (Feng et al., 2016).

## 6 Problems and prospects

The PB1 subunit of the influenza A virus RNA polymerase complex serves as the catalytic core of the RdRp. The lack of a proofreading mechanism is an intrinsic feature of the entire heterotrimeric complex (PB1–PB2–PA), resulting in a high nucleotide mismatch rate during viral RNA replication. Although this elevated error rate does not directly cause antigenic drift, it generates genetic diversity that drives viral adaptation and long-term evolution. Most existing studies have focused on the immune epitope variations of surface glycoproteins (HA), while insufficient attention has been paid to the driving role of PB1 host adaptive mutations in viral evolution. The origins of the influenza pandemics in 1918, 1957, and 1968 all involved avian reassortment and site mutations of the PB1 gene, which increased the adaptability of the virus (Williams et al., 2024; Plant et al., 2012). Therefore, continuous monitoring of site mutations and genetic reassortment dynamics of the PB1 gene is of great value for early warning of influenza pandemics.

Under the current research background of structural biology, X-ray crystallography and cryo-electron microscopy techniques have initially constructed the static structural framework of PB1 protein. However, the conformational transformation path from the initiation to the extension stage during its transcription process, as well as the dynamic structural changes induced by interaction with host proteins, still need to be systematically clarified. In the future, by integrating dynamic analysis techniques such as single-molecule fluorescence resonance energy transfer (FRET) and hydrogen-deuterium exchange mass spectrometry (HDX-MS), the conformation changes of PB1 protein under different functional states can be deeply revealed, providing a more comprehensive perspective for understanding the molecular mechanism of viral RNA synthesis.

**Figure 1:**
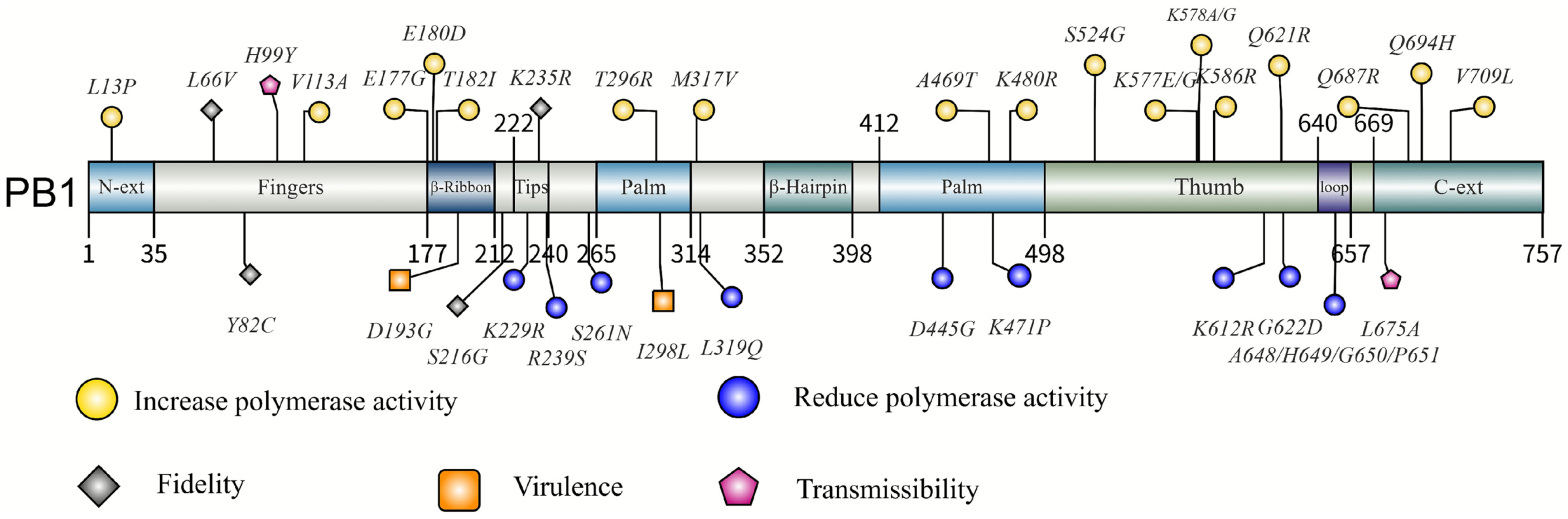
Mutation sites of influenza A virus PB1. Mutations in upper positions enhance function, while those in lower positions reduce function.

Vaccination is still the most effective strategy for preventing influenza infections (Sah et al., 2019). Site-specific mutations in the PB1 protein of influenza A virus may offer valuable insights for LAIV development, particularly in terms of enhancing viral stability and reducing the risk of reversion. The duration of protection and overall efficacy of current influenza vaccines remain suboptimal, driving a strategic shift in clinical research toward the development of next-generation candidates, with a particular focus on universal influenza vaccines (Trombetta et al., 2022; Chen et al., 2021). PB1 serves as a promising adjunct in a multivalent vaccine cocktail, enhancing the breadth of cross-protective immunity against heterosubtypic influenza variants (Uddbäck et al., 2016). Figure 1 illustrates the mutation sites in influenza A virus PB1. Mutations in upper domains are associated with enhanced polymerase activity, whereas those in lower domains correlate with impaired function. In the future, with the cross-integration of multi-disciplinary methods such as structural biology, molecular biology and computational biology, it may be possible to integrate multi-omics data to construct a functional prediction model for PB1 site mutations, build a dynamic monitoring system based on the functional hotspots of polymerase, and develop a universal vaccine platform capable of simulating natural immune processes. Research on PB1 will continue to drive innovation in influenza prevention and control strategies, providing an important scientific basis for addressing the public health challenges posed by influenza viruses.

## References

[ref1] Al Farroukh M. Kiseleva I. Bazhenova E. Stepanova E. Puchkova L. Rudenko L. (2022). Understanding the variability of certain biological properties of H1N1pdm09 influenza viruses. Vaccine 10:395. doi: 10.3390/vaccines10030395, 35335027 PMC8954537

[ref2] Almeida F. Santos L. A. Trigueiro-Louro J. M. Rebelo-de-Andrade H. (2022). Optimization of a(H1N1)pdm09 vaccine seed viruses: the source of PB1 and HA vRNA as a major determinant for antigen yield. Virus Res. 315:198795. doi: 10.1016/j.virusres.2022.198795, 35504447

[ref3] Arai Y. Kawashita N. Daidoji T. Ibrahim M. S. El-Gendy E. M. Takagi T. . (2016). Novel polymerase gene mutations for human adaptation in clinical isolates of avian H5N1 influenza viruses. PLoS Pathog. 12:e1005583. doi: 10.1371/journal.ppat.1005583, 27097026 PMC4838241

[ref4] Bai Y. Lei H. Song W. Shin S. C. Wang J. Xiao B. . (2024). Amino acids in the polymerase complex of shorebird-isolated H1N1 influenza virus impact replication and host-virus interactions in mammalian models. Emerg Microbes Infect. 13:2332652. doi: 10.1080/22221751.2024.2332652, 38517705 PMC11018082

[ref5] Baranovich T. Wong S. S. Armstrong J. Marjuki H. Webby R. J. Webster R. G. . (2013). T-705 (favipiravir) induces lethal mutagenesis in influenza A H1N1 viruses in vitro. J. Virol. 87, 3741–3751. doi: 10.1128/jvi.02346-12, 23325689 PMC3624194

[ref6] Boivin S. Cusack S. Ruigrok R. W. Hart D. J. (2010). Influenza A virus polymerase: structural insights into replication and host adaptation mechanisms. J. Biol. Chem. 285, 28411–28417. doi: 10.1074/jbc.R110.117531, 20538599 PMC2937865

[ref7] Bosaeed M. Kumar D. (2018). Seasonal influenza vaccine in immunocompromised persons. Hum. Vaccin. Immunother. 14, 1311–1322. doi: 10.1080/21645515.2018.1445446, 29485353 PMC6037456

[ref8] Brüssow H. (2022). The beginning and ending of a respiratory viral pandemic-lessons from the Spanish flu. Microb. Biotechnol. 15, 1301–1317. doi: 10.1111/1751-7915.14053, 35316560 PMC9049621

[ref9] Carter T. Iqbal M. (2024). The influenza A virus replication cycle: a comprehensive review. Viruses 16:316. doi: 10.3390/v16020316, 38400091 PMC10892522

[ref10] Chan J. J. Tang Y. S. Lo C. Y. Shaw P. C. (2023). Functional importance of the hydrophobic residue 362 in influenza A PB1 subunit. Viruses 15:396. doi: 10.3390/v15020396, 36851609 PMC9967172

[ref11] Chen L. L. Ip J. D. Chan W. M. Lam S. J. Leung R. C. Yip C. C. . (2025). Enhanced replication of a contemporary avian influenza A H9N2 virus in human respiratory organoids. Emerg. Microbes Infect. 14:2576574. doi: 10.1080/22221751.2025.2576574, 41099079 PMC12584838

[ref12] Chen J. Wang J. Zhang J. Ly H. (2021). Advances in development and application of influenza vaccines. Front. Immunol. 12:711997. doi: 10.3389/fimmu.2021.711997, 34326849 PMC8313855

[ref13] Cheung P. P. H. Watson S. J. Choy K.-T. Fun Sia S. Wong D. D. Y. Poon L. L. M. . (2014). Generation and characterization of influenza A viruses with altered polymerase fidelity. Nat. Commun. 5:4794. doi: 10.1038/ncomms5794, 25183443 PMC4155405

[ref14] Chin A. W. H. Yen H.-L. Krauss S. Webby R. J. Poon L. L. M. (2016). Recombinant influenza virus with a pandemic H2N2 polymerase complex has a higher adaptive potential than one with seasonal H2N2 polymerase complex. J. Gen. Virol. 97, 611–619. doi: 10.1099/jgv.0.000385, 26703222 PMC4804605

[ref15] Cobbin J. C. Verity E. E. Gilbertson B. P. Rockman S. P. Brown L. E. (2013). The source of the PB1 gene in influenza vaccine reassortants selectively alters the hemagglutinin content of the resulting seed virus. J. Virol. 87, 5577–5585. doi: 10.1128/jvi.02856-12, 23468502 PMC3648160

[ref16] Cowling B. J. Okoli G. N. (2024). Influenza vaccine effectiveness and progress towards a universal influenza vaccine. Drugs 84, 1013–1023. doi: 10.1007/s40265-024-02083-8, 39167316 PMC11438668

[ref17] Cox A. Dewhurst S. Lyles D. S. (2016). A single mutation at PB1 residue 319 dramatically increases the safety of PR8 live attenuated influenza vaccine in a murine model without compromising vaccine efficacy. J. Virol. 90, 2702–2705. doi: 10.1128/jvi.02723-15 PMC4810719 26676793

[ref18] Cox A. Schmierer J. D'Angelo J. Smith A. Levenson D. Treanor J. . (2020). A mutated PB1 residue 319 synergizes with the PB2 N265S mutation of the live attenuated influenza vaccine to convey temperature sensitivity. Viruses 12:1246. doi: 10.3390/v12111246 33142846 PMC7693792

[ref19] Dreier C. Resa-Infante P. Thiele S. Stanelle-Bertram S. Walendy-Gnirß K. Speiseder T. . (2019). Mutations in the H7 HA and PB1 genes of avian influenza A viruses increase viral pathogenicity and contact transmission in guinea pigs. Emerg. Microbes Infect. 8, 1324–1336. doi: 10.1080/22221751.2019.1663131, 31503518 PMC6746284

[ref20] Du Y. Wu N. C. Jiang L. Zhang T. Gong D. Shu S. . (2016). Annotating protein functional residues by coupling high-throughput fitness profile and homologous-structure analysis. mBio 7:e01801. doi: 10.1128/mBio.01801-16, 27803181 PMC5090041

[ref21] Elshina E. Te Velthuis A. J. W. (2021). The influenza virus RNA polymerase as an innate immune agonist and antagonist. Cell. Mol. Life Sci. 78, 7237–7256. doi: 10.1007/s00018-021-03957-w, 34677644 PMC8532088

[ref22] Fan H. Walker A. P. Carrique L. Keown J. R. Serna Martin I. Karia D. . (2019). Structures of influenza A virus RNA polymerase offer insight into viral genome replication. Nature 573, 287–290. doi: 10.1038/s41586-019-1530-7, 31485076 PMC6795553

[ref23] Feng X. Wang Z. Shi J. Deng G. Kong H. Tao S. . (2016). Glycine at position 622 in PB1 contributes to the virulence of H5N1 avian influenza virus in mice. J. Virol. 90, 1872–1879. doi: 10.1128/jvi.02387-15, 26656683 PMC4733975

[ref24] Fodor E. Te Velthuis A. J. W. (2020). Structure and function of the influenza virus transcription and replication machinery. Cold Spring Harb. Perspect. Med. 10. doi: 10.1101/cshperspect.a038398, 31871230 PMC7334866

[ref25] Gíria M. Rebelo de Andrade H. (2014). Genetic evolution of PB1 in the zoonotic transmission of influenza A(H1) virus. Infect. Genet. Evol. 27, 234–243. doi: 10.1016/j.meegid.2014.07.024, 25092558

[ref26] Gíria M. Santos L. Louro J. Rebelo de Andrade H. (2016). Reverse genetics vaccine seeds for influenza: proof of concept in the source of PB1 as a determinant factor in virus growth and antigen yield. Virology 496, 21–27. doi: 10.1016/j.virol.2016.05.015, 27240145

[ref27] Goldhill D. H. Te Velthuis A. J. W. Fletcher R. A. Langat P. Zambon M. Lackenby A. . (2018). The mechanism of resistance to favipiravir in influenza. Proc. Natl. Acad. Sci. USA 115, 11613–11618. doi: 10.1073/pnas.1811345115, 30352857 PMC6233120

[ref28] Goldhill D. H. Yan A. Frise R. Zhou J. Shelley J. Gallego Cortés A. . (2021). Favipiravir-resistant influenza A virus shows potential for transmission. PLoS Pathog. 17:e1008937. doi: 10.1371/journal.ppat.1008937, 34061908 PMC8195362

[ref29] Grohskopf L. A. Blanton L. H. Ferdinands J. M. Reed C. Dugan V. G. Daskalakis D. C. (2025). Prevention and control of seasonal influenza with vaccines: recommendations of the advisory committee on immunization practices - United States, 2025-26 influenza season. MMWR Morb. Mortal Wkly. Rep. 74, 500–507. doi: 10.15585/mmwr.mm7432a2, 40879559 PMC12393693

[ref30] Günl F. Krischuns T. Schreiber J. A. Henschel L. Wahrenburg M. Drexler H. C. A. . (2023). The ubiquitination landscape of the influenza A virus polymerase. Nat. Commun. 14:787. doi: 10.1038/s41467-023-36389-0, 36774438 PMC9922279

[ref31] Guo Y. Sun T. Bai X. Liang B. Deng L. Zheng Y. . (2023). Comprehensive analysis of the key amino acid substitutions in the polymerase and NP of avian influenza virus that enhance polymerase activity and affect adaptation to mammalian hosts. Vet. Microbiol. 282:109760. doi: 10.1016/j.vetmic.2023.109760 37120967

[ref32] Hannoun C. (2013). The evolving history of influenza viruses and influenza vaccines. Expert Rev. Vaccines 12, 1085–1094. doi: 10.1586/14760584.2013.824709, 24024871

[ref33] Houser K. Subbarao K. (2015). Influenza vaccines: challenges and solutions. Cell Host Microbe 17, 295–300. doi: 10.1016/j.chom.2015.02.012, 25766291 PMC4362519

[ref34] Joshi S. Parkar J. Ansari A. Vora A. Talwar D. Tiwaskar M. . (2021). Role of favipiravir in the treatment of COVID-19. Int. J. Infect. Dis. 102, 501–508. doi: 10.1016/j.ijid.2020.10.069, 33130203 PMC7831863

[ref35] Kamiki H. Matsugo H. Kobayashi T. Ishida H. Takenaka-Uema A. Murakami S. . (2018). A PB1-K577E mutation in H9N2 influenza virus increases polymerase activity and pathogenicity in mice. Viruses 10:653. doi: 10.3390/v10110653, 30463209 PMC6266086

[ref36] Keown J. R. Zhu Z. Carrique L. Fan H. Walker A. P. Serna Martin I. . (2022). Mapping inhibitory sites on the RNA polymerase of the 1918 pandemic influenza virus using nanobodies. Nat. Commun. 13:251. doi: 10.1038/s41467-021-27950-w, 35017564 PMC8752864

[ref37] Kim Y. H. Hong K. J. Kim H. Nam J. H. (2022). Influenza vaccines: past, present, and future. Rev. Med. Virol. 32:e2243. doi: 10.1002/rmv.2243, 33949021 PMC8209895

[ref38] Klein S. L. Siegers J. Y. Ferreri L. Eggink D. Veldhuis Kroeze E. J. B. te Velthuis A. J. W. . (2023). Evolution of highly pathogenic H5N1 influenza A virus in the central nervous system of ferrets. PLoS Pathog. 19:e1011214. doi: 10.1371/journal.ppat.1011214 36897923 PMC10032531

[ref39] Kouba T. Drncová P. Cusack S. (2019). Structural snapshots of actively transcribing influenza polymerase. Nat. Struct. Mol. Biol. 26, 460–470. doi: 10.1038/s41594-019-0232-z, 31160782 PMC7610713

[ref40] Krammer F. Smith G. J. D. Fouchier R. A. M. Peiris M. Kedzierska K. Doherty P. C. . (2018). Influenza. Nat. Rev. Dis. Primers 4:3. doi: 10.1038/s41572-018-0002-y, 29955068 PMC7097467

[ref41] Krischuns T. Lukarska M. Naffakh N. Cusack S. (2021). Influenza virus RNA-dependent RNA polymerase and the host transcriptional apparatus. Annu. Rev. Biochem. 90, 321–348. doi: 10.1146/annurev-biochem-072820-100645 33770447

[ref42] Li Y. Arcos S. Sabsay K. R. Te Velthuis A. J. W. Lauring A. S. (2023). Deep mutational scanning reveals the functional constraints and evolutionary potential of the influenza A virus PB1 protein. J. Virol. 97:e0132923. doi: 10.1128/jvi.01329-23, 37882522 PMC10688322

[ref43] Li O. T. Chan M. C. Leung C. S. Chan R. W. Guan Y. Nicholls J. M. . (2009). Full factorial analysis of mammalian and avian influenza polymerase subunits suggests a role of an efficient polymerase for virus adaptation. PLoS One 4:e5658. doi: 10.1371/journal.pone.0005658, 19462010 PMC2680953

[ref44] Li K. S. Guan Y. Wang J. Smith G. J. Xu K. M. Duan L. . (2004). Genesis of a highly pathogenic and potentially pandemic H5N1 influenza virus in eastern Asia. Nature 430, 209–213. doi: 10.1038/nature02746, 15241415

[ref45] Li J. Jin Y. Wang Y. Shang L. Cao B. (2025). New developments in influenza polymerase inhibitors. J. Infect. Dis. 232, S227–S242. doi: 10.1093/infdis/jiaf126, 41102615

[ref46] Li J. Liang L. Jiang L. Wang Q. Wen X. Zhao Y. . (2021). Viral RNA-binding ability conferred by SUMOylation at PB1 K612 of influenza A virus is essential for viral pathogenesis and transmission. PLoS Pathog. 17:e1009336. doi: 10.1371/journal.ppat.1009336, 33571308 PMC7904188

[ref47] Li H. Wu Y. Li M. Guo L. Gao Y. Wang Q. . (2023). An intermediate state allows influenza polymerase to switch smoothly between transcription and replication cycles. Nat. Struct. Mol. Biol. 30, 1183–1192. doi: 10.1038/s41594-023-01043-2, 37488357

[ref48] Li M. Wu Y. Li H. Song W. Chen Z. Peng Y. . (2025). Mutagenesis studies suggest a mechanism for influenza polymerase stalling during polyadenylation. Nucleic Acids Res. 53:gkae1225. doi: 10.1093/nar/gkae1225, 39676676 PMC11797019

[ref49] Lin R.-W. Chen G.-W. Sung H.-H. Lin R.-J. Yen L.-C. Tseng Y.-L. . (2019). Naturally occurring mutations in PB1 affect influenza A virus replication fidelity, virulence, and adaptability. J. Biomed. Sci. 26:55. doi: 10.1186/s12929-019-0547-4, 31366399 PMC6668090

[ref50] Ling Y. H. Wang H. Han M. Q. Wang D. Hu Y. X. Zhou K. . (2022). Nucleoporin 85 interacts with influenza A virus PB1 and PB2 to promote its replication by facilitating nuclear import of ribonucleoprotein. Front. Microbiol. 13:895779. doi: 10.3389/fmicb.2022.895779, 36051755 PMC9426659

[ref51] Linster M. van Boheemen S. de Graaf M. Schrauwen E. J. A. Lexmond P. Mänz B. . (2014). Identification, characterization, and natural selection of mutations driving airborne transmission of a/H5N1 virus. Cell 157, 329–339. doi: 10.1016/j.cell.2014.02.040, 24725402 PMC4003409

[ref52] Lowen A. C. Chen K.-Y. Santos Afonso E. D. Enouf V. Isel C. Naffakh N. (2019). Influenza virus polymerase subunits co-evolve to ensure proper levels of dimerization of the heterotrimer. PLoS Pathog. 15:e1008034. doi: 10.1371/journal.ppat.1008034 31581279 PMC6776259

[ref53] Mori K. Ohniwa R. L. Takizawa N. Naito T. Saito M. Schultz-Cherry S. (2021). Development of a genetically stable live attenuated influenza vaccine strain using an engineered high-fidelity viral polymerase. J. Virol. 95:e00493. doi: 10.1128/jvi.00493-21 33827947 PMC8316084

[ref54] Naito T. Mori K. Ushirogawa H. Takizawa N. Nobusawa E. Odagiri T. . (2017). Generation of a genetically stable high-fidelity influenza vaccine strain. J. Virol. 91:e01073. doi: 10.1128/jvi.01073-16 28053101 PMC5331824

[ref55] Naito T. Shirai K. Mori K. Muratsu H. Ushirogawa H. Ohniwa R. L. . (2019). Tyr82 amino acid mutation in PB1 polymerase induces an influenza virus mutator phenotype. J. Virol. 93:e00834. doi: 10.1128/jvi.00834-19, 31462570 PMC6819930

[ref56] Naito T. Ushirogawa H. Kunishio M. Yano H. Saito S. Higeuchi T. . (2025). Live-attenuated influenza virus vaccine strain with an engineered temperature-sensitive and genetically stable viral polymerase variant. J. Virol. 99:e0139025. doi: 10.1128/jvi.01390-25, 41231009 PMC12724131

[ref57] Noda T. Kawaoka Y. (2010). Structure of influenza virus ribonucleoprotein complexes and their packaging into virions. Rev. Med. Virol. 20, 380–391. doi: 10.1002/rmv.666, 20853340 PMC6029254

[ref58] Nogales A. Steel J. Liu W. C. Lowen A. C. Rodriguez L. Chiem K. . (2022). Mutation L319Q in the PB1 polymerase subunit improves attenuation of a candidate live-attenuated influenza A virus vaccine. Microbiol Spectr. 10:e0007822. doi: 10.1128/spectrum.00078-22, 35583364 PMC9241597

[ref59] Oymans J. te Velthuis A. J. W. Schultz-Cherry S. (2018). A mechanism for priming and realignment during influenza A virus replication. J. Virol. 92:e01773. doi: 10.1128/jvi.01773-17 29118119 PMC5774886

[ref60] Pauly M. D. Lyons D. M. Fitzsimmons W. J. Lauring A. S. (2017). Epistatic interactions within the influenza A virus polymerase complex mediate mutagen resistance and replication fidelity. mSphere 2:e00323. doi: 10.1128/mSphere.00323-17, 28815216 PMC5557677

[ref61] Peacock T. P. Sheppard C. M. Staller E. Barclay W. S. (2019). Host determinants of influenza RNA synthesis. Annu. Rev. Virol. 6, 215–233. doi: 10.1146/annurev-virology-092917-043339, 31283439

[ref62] Peng R. Xu X. Nepal B. Gong Y. Li F. Ferretti M. B. . (2025). Molecular basis of influenza ribonucleoprotein complex assembly and processive RNA synthesis. Science 388:eadq7597. doi: 10.1126/science.adq7597, 40373132 PMC12240686

[ref63] Pflug A. Guilligay D. Reich S. Cusack S. (2014). Structure of influenza A polymerase bound to the viral RNA promoter. Nature 516, 355–360. doi: 10.1038/nature14008 25409142

[ref64] Pflug A. Lukarska M. Resa-Infante P. Reich S. Cusack S. (2017). Structural insights into RNA synthesis by the influenza virus transcription-replication machine. Virus Res. 234, 103–117. doi: 10.1016/j.virusres.2017.01.013, 28115197

[ref65] Plant E. P. Liu T. M. Xie H. Ye Z. (2012). Mutations to a/Puerto Rico/8/34 PB1 gene improves seasonal reassortant influenza A virus growth kinetics. Vaccine 31, 207–212. doi: 10.1016/j.vaccine.2012.10.060, 23116694

[ref66] Poon L. L. M. Chu C. Fan S. Li C. Macken C. Kim J. H. . (2012). Functional analysis of conserved motifs in influenza virus PB1 protein. PLoS One 7:e36113. doi: 10.1371/journal.pone.0036113 22615752 PMC3352917

[ref67] Ran W. Schön J. Ciminski K. Kraft J. Kessler S. Euchner S. . (2022). Generation of an attenuated chimeric bat influenza A virus live-vaccine prototype. Microbiol Spectr. 10:e0142422. doi: 10.1128/spectrum.01424-22, 36445145 PMC9769755

[ref68] Raymond D. D. Stewart S. M. Lee J. Ferdman J. Bajic G. Do K. T. . (2016). Influenza immunization elicits antibodies specific for an egg-adapted vaccine strain. Nat. Med. 22, 1465–1469. doi: 10.1038/nm.4223, 27820604 PMC5485662

[ref69] Reuther P. Mänz B. Brunotte L. Schwemmle M. Wunderlich K. (2011). Targeting of the influenza A virus polymerase PB1-PB2 interface indicates strain-specific assembly differences. J. Virol. 85, 13298–13309. doi: 10.1128/jvi.00868-11, 21957294 PMC3233147

[ref70] Ryu S. Cowling B. J. (2021). Human influenza epidemiology. Cold Spring Harb. Perspect. Med. 11. doi: 10.1101/cshperspect.a038356, 32988982 PMC8634793

[ref71] Sah P. Alfaro-Murillo J. A. Fitzpatrick M. C. Neuzil K. M. Meyers L. A. Singer B. H. . (2019). Future epidemiological and economic impacts of universal influenza vaccines. Proc. Natl. Acad. Sci. USA 116, 20786–20792. doi: 10.1073/pnas.1909613116, 31548402 PMC6789917

[ref72] Samantaray M. Pushan S. S. Rajagopalan M. Ramaswamy A. (2023). Structural dynamics of the RNA dependent RNA polymerase of H1N1 strain affecting humans: a bioinformatics approach. J. Biomol. Struct. Dyn. 42, 10876–10889. doi: 10.1080/07391102.2023.2259481, 37728538

[ref73] Santos L. A. Almeida F. Gíria M. Trigueiro-Louro J. Rebelo-de-Andrade H. (2023). Adaptive evolution of PB1 from influenza A(H1N1)pdm09 virus towards an enhanced fitness. Virology 578, 1–6. doi: 10.1016/j.virol.2022.11.003, 36423573

[ref74] Scott J. Abers M. S. Marwah H. K. McCann N. C. Meyerowitz E. A. Richterman A. . (2025). Updated evidence for Covid-19, RSV, and influenza vaccines for 2025-2026. N. Engl. J. Med. 393, 2221–2242. doi: 10.1056/NEJMsa2514268, 41160817

[ref75] Slaine P. D. MacRae C. Kleer M. Lamoureux E. McAlpine S. Warhuus M. . (2018). Adaptive mutations in influenza A/California/07/2009 enhance polymerase activity and infectious virion production. Viruses 10:272. doi: 10.3390/v10050272, 29783694 PMC5977265

[ref76] Stubbs T. M. Te Velthuis A. J. (2014). The RNA-dependent RNA polymerase of the influenza A virus. Future Virol. 9, 863–876. doi: 10.2217/fvl.14.66, 25431616 PMC4243023

[ref77] Su W. Harfoot R. Su Y. C. F. DeBeauchamp J. Joseph U. Jayakumar J. . (2021). Ancestral sequence reconstruction pinpoints adaptations that enable avian influenza virus transmission in pigs. Nat. Microbiol. 6, 1455–1465. doi: 10.1038/s41564-021-00976-y, 34702977 PMC8557130

[ref78] Sun T. Guo Y. Zhao L. Fan M. Huang N. Tian M. . (2021). Evolution of the PB1 gene of human influenza A (H3N2) viruses circulating between 1968 and 2019. Transbound. Emerg. Dis. 69, 1824–1836. doi: 10.1111/tbed.14161, 34033262

[ref79] Taubenberger J. K. Reid A. H. Lourens R. M. Wang R. Jin G. Fanning T. G. (2005). Characterization of the 1918 influenza virus polymerase genes. Nature 437, 889–893. doi: 10.1038/nature04230, 16208372

[ref80] Te Velthuis A. J. W. Grimes J. M. Fodor E. (2021). Structural insights into RNA polymerases of negative-sense RNA viruses. Nat. Rev. Microbiol. 19, 303–318. doi: 10.1038/s41579-020-00501-8, 33495561 PMC7832423

[ref81] Te Velthuis A. J. W. Oymans J. (2018). Initiation, elongation, and realignment during influenza virus mRNA synthesis. J. Virol. 92:e01775. doi: 10.1128/jvi.01775-17, 29142123 PMC5774887

[ref82] Te Velthuis A. J. W. Robb N. C. Kapanidis A. N. Fodor E. (2016). The role of the priming loop in influenza A virus RNA synthesis. Nat. Microbiol. 1:16029. doi: 10.1038/nmicrobiol.2016.29, 27572643

[ref83] Trombetta C. M. Kistner O. Montomoli E. Viviani S. Marchi S. (2022). Influenza viruses and vaccines: the role of vaccine effectiveness studies for evaluation of the benefits of influenza vaccines. Vaccine 10:714. doi: 10.3390/vaccines10050714, 35632470 PMC9143275

[ref84] Turnbull M. L. Wang Y. Clare S. Lieber G. Williams S. L. Noerenberg M. . (2025). Avian-origin influenza A viruses tolerate elevated pyrexic temperatures in mammals. Science 390:eadq4691. doi: 10.1126/science.adq4691, 41308154 PMC7618609

[ref85] Uchikubo-Kamo T. Ishimoto N. Umezawa H. Hirohama M. Ono M. Kawabata H. . (2024). Structural insights into influenza A virus RNA polymerase PB1 binding to nuclear import host factor RanBP5. Biochem. Biophys. Res. Commun. 739:150952. doi: 10.1016/j.bbrc.2024.150952, 39536408

[ref86] Uddbäck I. E. Steffensen M. A. Pedersen S. R. Nazerai L. Thomsen A. R. Christensen J. P. (2016). PB1 as a potential target for increasing the breadth of T-cell mediated immunity to influenza A. Sci. Rep. 6:35033. doi: 10.1038/srep35033, 27713532 PMC5054373

[ref87] Wachsmuth-Melm M. Peterl S. O'Riain A. Makroczyová J. Fischer K. Krischuns T. . (2025). Visualizing influenza A virus assembly by in situ cryo-electron tomography. Nat. Commun. 16:9394. doi: 10.1038/s41467-025-65117-z, 41130956 PMC12550032

[ref88] Walker A. P. Fodor E. (2019). Interplay between influenza virus and the host RNA polymerase II transcriptional machinery. Trends Microbiol. 27, 398–407. doi: 10.1016/j.tim.2018.12.013, 30642766 PMC6467242

[ref89] Wandzik J. M. Kouba T. Cusack S. (2021). Structure and function of influenza polymerase. Cold Spring Harb. Perspect. Med. 11. doi: 10.1101/cshperspect.a038372, 32341065 PMC8415296

[ref90] Wandzik J. M. Kouba T. Karuppasamy M. Pflug A. Drncova P. Provaznik J. . (2020). A structure-based model for the complete transcription cycle of influenza polymerase. Cell 181, 877–893.e21. doi: 10.1016/j.cell.2020.03.061, 32304664

[ref91] Wang F. Liu G. Lu Y. Hlasny M. Liu Q. Zhou Y. (2020). Acquisition of avian-origin PB1 facilitates viral RNA synthesis by the 2009 pandemic H1N1 virus polymerase. Viruses 12:266. doi: 10.3390/v12030266, 32121117 PMC7150768

[ref92] Wang X. Tang X. E. Zheng H. Gao R. Lu X. Yang W. . (2024). Amino acid mutations PB1-V719M and PA-N444D combined with PB2-627K contribute to the pathogenicity of H7N9 in mice. Vet. Res. 55:86. doi: 10.1186/s13567-024-01342-6, 38970119 PMC11227215

[ref93] Waters K. Wan H. J. Han L. Xue J. Ykema M. Tao Y. J. . (2021). Variations outside the conserved motifs of PB1 catalytic active site may affect replication efficiency of the RNP complex of influenza A virus. Virology 559, 145–155. doi: 10.1016/j.virol.2021.04.001, 33887645 PMC8579824

[ref94] Wei K. Sun H. Sun Z. Sun Y. Kong W. Pu J. . (2014). Influenza A virus acquires enhanced pathogenicity and transmissibility after serial passages in swine. J. Virol. 88, 11981–11994. doi: 10.1128/jvi.01679-14, 25100840 PMC4178715

[ref95] Welkers M. R. A. Pawestri H. A. Fonville J. M. Sampurno O. D. Pater M. Holwerda M. . (2019). Genetic diversity and host adaptation of avian H5N1 influenza viruses during human infection. Emerg Microbes Infect. 8, 262–271. doi: 10.1080/22221751.2019.1575700, 30866780 PMC6455201

[ref96] Wendel I. Rubbenstroth D. Doedt J. Kochs G. Wilhelm J. Staeheli P. . (2015). The avian-origin PB1 gene segment facilitated replication and transmissibility of the H3N2/1968 pandemic influenza virus. J. Virol. 89, 4170–4179. doi: 10.1128/jvi.03194-14, 25631088 PMC4442368

[ref97] Williams S. L. Qi L. Sheng Z. M. Xiao Y. Freeman A. Matthews L. . (2024). Effect of pandemic influenza A virus PB1 genes of avian origin on viral RNA polymerase activity and pathogenicity. Sci. Adv. 10:eads5735. doi: 10.1126/sciadv.ads5735, 39671482 PMC11641000

[ref98] World Health Organization. Influenza situation updates 2025 (2025) Available online at: https://www.who.int/health-topics/influenza-seasonal#tab=tab_1 (Accessed June 28, 2025).

[ref99] Worobey M. Han G. Z. Rambaut A. (2014). Genesis and pathogenesis of the 1918 pandemic H1N1 influenza A virus. Proc. Natl. Acad. Sci. USA 111, 8107–8112. doi: 10.1073/pnas.1324197111, 24778238 PMC4050607

[ref100] Xu C. Hu W. B. Xu K. He Y. X. Wang T. Y. Chen Z. . (2012). Amino acids 473V and 598P of PB1 from an avian-origin influenza A virus contribute to polymerase activity, especially in mammalian cells. J. Gen. Virol. 93, 531–540. doi: 10.1099/vir.0.036434-0, 22090209

[ref101] Xu X. Zhang L. Chu J. T. S. Wang Y. Chin A. W. H. Chong T. H. . (2021). A novel mechanism of enhanced transcription activity and fidelity for influenza A viral RNA-dependent RNA polymerase. Nucleic Acids Res. 49, 8796–8810. doi: 10.1093/nar/gkab660, 34379778 PMC8421151

[ref102] Xue R. Ma H. Jiang Z. Xing L. Wang G. Lan Z. . (2025). Diversity of the H9N2 avian influenza virus in Shandong Province, China. Transbound. Emerg. Dis. 2025:1432483. doi: 10.1155/tbed/1432483, 40302737 PMC12016696

[ref103] Youk S.-S. Leyson C. M. Seibert B. A. Jadhao S. Perez D. R. Suarez D. L. . (2021). Mutations in PB1, NP, HA, and NA contribute to increased virus fitness of H5N2 highly pathogenic avian influenza virus clade 2.3.4.4 in chickens. J. Virol. 95:e01675. doi: 10.1128/jvi.01675-20, 33268526 PMC8092828

[ref104] Yu Z. Cheng K. Sun W. Zhang X. Li Y. Wang T. . (2015). A PB1 T296R substitution enhance polymerase activity and confer a virulent phenotype to a 2009 pandemic H1N1 influenza virus in mice. Virology 486, 180–186. doi: 10.1016/j.virol.2015.09.014, 26453960

[ref105] Zhang J. Hu Y. Wu N. Wang J. (2020). Discovery of influenza polymerase PA-PB1 interaction inhibitors using an in vitro Split-luciferase complementation-based assay. ACS Chem. Biol. 15, 74–82. doi: 10.1021/acschembio.9b00552, 31714745 PMC7028398

[ref106] Zhang X. Li Y. Jin S. Zhang Y. Sun L. Hu X. . (2021). PB1 S524G mutation of wild bird-origin H3N8 influenza A virus enhances virulence and fitness for transmission in mammals. Emerg. Microbes Infect. 10, 1038–1051. doi: 10.1080/22221751.2021.1912644, 33840358 PMC8183522

[ref107] Zhou B. Meliopoulos V. A. Wang W. Lin X. Stucker K. M. Halpin R. A. . (2016). Reversion of cold-adapted live attenuated influenza vaccine into a pathogenic virus. J. Virol. 90, 8454–8463. doi: 10.1128/jvi.00163-16, 27440882 PMC5021423

[ref108] Zhu Z. Fodor E. Keown J. R. (2023). A structural understanding of influenza virus genome replication. Trends Microbiol. 31, 308–319. doi: 10.1016/j.tim.2022.09.015, 36336541

